# Integrated single-cell multi-omics analysis delineates neonatal immune dysregulation associated with gestational diabetes mellitus

**DOI:** 10.3389/fimmu.2026.1924940

**Published:** 2026-09-30

**Authors:** Renke He, Zhipeng Liu, Qingju Meng, Kaiyue Wu, Junwei Wu, Yanqiu Li, Oscar Junhong Luo, Yangqiu Li

**Affiliations:** 1 Department of Hematology, First Affiliated Hospital of Jinan University, Guangzhou, China; 2 Shantou Central Hospital, Shantou Clinical Medical College of Jinan University, Shantou, Guangdong, China; 3 Key Laboratory for Regenerative Medicine of Ministry of Education, Institute of Hematology, School of Medicine, Jinan University, Guangzhou, China; 4 Department of Systems Biomedical Sciences, School of Medicine, Jinan University, Guangzhou, China; 5 Department of Internal Medicine, Huaxing Rehabilitation Hospital, Guangzhou, China; 6 Gynaecology and Obstetrics Department, Guangzhou Panyu District Maternal and Child Health Hospital, Guangzhou, Guangdong, China; 7 Department of Anesthesiology, The First Affiliated Hospital of Jinan University, School of Medicine, Jinan University, Guangzhou, China; 8 State Key Laboratory of Bioactive Molecules and Druggability Assessment, School of Medicine, Jinan University, Guangzhou, China

**Keywords:** gestational diabetes mellitus, immune repertoire, intrauterine hyperglycemia, neonatal immunity, single-cell RNA sequencing, single-cell TCR, Tpex-like cells

## Abstract

**Introduction:**

Gestational diabetes mellitus (GDM) is a common pregnancy complication which could induce immune dysfunction. Although intrauterine hyperglycemia impairs fetal immune development, its cell-type specific effects on neonatal immune cells, and cell‑cell communication remain unclear.

**Methods:**

In this pilot study, we performed integrated 5′ single‑cell RNA sequencing and TCR repertoire analysis of cord blood mononuclear cells from two GDM and one type 2 diabetes mellitus (T2DM) mother–neonate pairs, with public healthy cord blood data (GSE157007) as controls. After quality control, 50,656 cells were analyzed using Seurat, scRepertoire, SCENIC, Monocle 2, decoupleR, and CellChat.

**Results:**

Myeloid lineage expansion and reduced naive T‑cell proportions have been found in hyperglycemia‑exposed cord blood. TCR analysis revealed mild clonal diversity compression, a shift toward shorter CDR3 lengths, and fewer ultralong CDR3 sequences in hyperglycemia‑exposed cord blood. The Tpex‑like cell population, which co‑expresses stemness markers (TCF7, BACH2), early development markers of T cells (IKZF1, IKZF2) and the pre‑exhaustion transcription factor TOX, was enriched in cord blood from GDM and T2DM neonates. This subset displayed elevated activity of TGF-β, MAPK and PI3K signaling pathways and predominantly resided within unexpanded T cell clonotypes. Pseudotime trajectory profiling indicates that Tpex-like cells reside at an intermediate differentiation state between precursor and effector T‑cell populations. Cell communication analysis indicated increased immune interaction strength in GDM cord blood, with enrichment of pro‑inflammatory pathways (SEMA4, IL‑16, SIRP), whereas homeostatic pathways (LAIR1, GALECTIN) dominated in healthy controls.

**Discussion:**

We characterized multi-layered neonatal immune dysregulation observed in the setting of GDM‑associated intrauterine hyperglycemia. Tpex‑like cells represent a candidate cellular state linked to early immune changes in GDM offspring. We firstly identified candidate molecular signatures for subsequent mechanistic and translational exploration. These pilot findings require validation in larger independent cohorts.

## Introduction

Gestational diabetes mellitus (GDM) is one of the most common metabolic complications during pregnancy. Based on the diagnostic criteria of the International Association of Diabetes and Pregnancy Study Groups (IADPSG), its global prevalence is approximately 14%, and this number continues to rise with the obesity epidemic and the increasing proportion of advanced maternal age (1). GDM not only significantly increases the maternal risk of preeclampsia, cesarean section, and postpartum progression to type 2 diabetes mellitus (T2DM), but could also lead to long-term impact on offspring health, which all warrants serious attention. Epidemiological studies have demonstrated that offspring exposed to GDM have a significantly elevated risk of developing obesity, metabolic syndrome, T2DM, and allergic diseases during childhood and early adulthood (2), suggesting that intrauterine hyperglycemia exposure may exert lasting effects on multiple fetal organ systems through developmental programming mechanisms (3). The immune system serves as the core barrier for defending against pathogens and maintaining homeostasis. Its normal development during the embryonic and perinatal periods is crucial for lifelong immune health. However, the impact of intrauterine metabolic stress on this critical developmental window remains poorly understood.

Previous studies have indicated that the GDM intrauterine environment can affect the fetal immune system through various mechanisms. Sustained hyperglycemia can induce the accumulation of reactive oxygen species (ROS) and the generation of advanced glycation end products (AGEs), driving pro-inflammatory responses in myeloid immune cells by activating innate immune inflammatory pathways such as NF-κB. Meanwhile, the excessive secretion of pro-inflammatory cytokines (e.g., IL-6, TNF-α) by GDM placental trophoblasts and Hofbauer cells further exacerbates the intrauterine inflammatory microenvironment, exerting potential impacts on the fetal hematopoietic microenvironment and immune cell development (3). Conventional studies based on flow cytometry and bulk RNA sequencing have suggested an elevated proportion of innate immune cells and certain functional abnormalities in T cells within the cord blood mononuclear cells (CBMCs) of neonates born to GDM mothers (4). However, conventional flow cytometry is limited to cellular phenotyping without transcriptomic or clonal resolution, whereas bulk RNA-seq only averages heterogeneous signals and fails to detect rare cell subsets (40). Furthermore, paired comparative studies of immune cells between maternal peripheral blood mononuclear cells (PBMCs) and CBMCs in GDM are extremely limited, leaving the similarities, differences, and potential immune cross-talk between the maternal and infant immune compartments largely uncharacterized. In adaptive immunity, the diversity and clonal structure of the T-cell receptor (TCR) repertoire are critical indicators for evaluating antigen responsiveness. However, systematic investigations into how intrauterine hyperglycemic exposure shapes neonatal T cell lineage development and TCR repertoire architecture remain insufficiently characterized.

The rapid development of single-cell RNA sequencing (scRNA-seq) technology provides a powerful tool for systematically dissecting immune cell heterogeneity at single-cell resolution. Coupled with TCR repertoire sequencing, it is possible to simultaneously capture the transcriptomic profiles and clonotype information of individual T cells, allowing for a comprehensive delineation of T-cell immune responses from both functional states and immune repertoire dimensions. In recent years, scRNA-seq has been widely applied in reproductive immunology research; for instance, Vento-Tormo et al. (5) mapped a large-scale single-cell transcriptomic atlas of the early human maternal-fetal interface, systematically revealing the cell composition and maternal-fetal communication networks of the decidual immune microenvironment. Since then, scRNA-seq has yielded significant advances in unraveling the immune mechanisms underlying placental development and pregnancy complications such as preeclampsia. Single-cell transcriptomics has also been applied to dissect early hematopoietic developmental transitions, including endothelial-to-hematopoietic specification events in the yolk sac (37), underscoring the broader utility of this approach for resolving fetal immune ontogeny at single-cell resolution.

However, comprehensive single-cell transcriptomic studies focusing on paired maternal PBMCs and CBMCs, as well as comparisons between GDM and healthy CBMC immune cells, remain scarce. In particular, there is a lack of in-depth exploration regarding T-cell lineage composition and functional states, TCR repertoire diversity, and the remodeling of intercellular communication networks under GDM conditions. To address these gaps, we combined scRNA‑seq and scTCR‑seq to generate an integrated immune atlas covering paired maternal‑neonatal PBMC‑CBMC samples from hyperglycaemic‑exposed and healthy pregnancies. We evaluated changes in the diversity and clonality of neonatal T‑cell repertoires linked to intrauterine hyperglycaemic exposure. Through gene sets scoring and canonical pathway activity profiling, we identified a Tpex‑like subset enriched among rare neonatal T‑cell clones, whose transcriptional profile resembles canonical Tpex cells, with a TCF7‑ and FOXP1‑dominated regulatory network. Pseudotime analysis placed this subset at an intermediate differentiation state between precursor and effector T cells. Finally, we uncovered correlated shifts in intercellular immune signaling, revealing potential mechanistic links among TCR‑repertoire remodeling, Tpex‑like cell states and altered immune crosstalk. Our single-cell data characterizes candidate early immune alterations in GDM neonates, and provides correlative molecular signatures to guide further research on offspring long-term immune susceptibility.

## Results

### Altered immune cell landscape between mothers and hyperglycemia-exposed neonates

To profile immune landscapes under maternal hyperglycemia, we conducted paired 5′ scRNA-seq and TCR sequencing on PBMC-CBMC dyads from GDM and T2DM pregnancies, with GSE157007 cord blood data serving as healthy controls (**Supplementary Table S1**; **Figure 1a**). After standardized quality filtering and Harmony-based batch correction, cross-dataset cell-type distributions showed no residual batch effects outside the granulocyte compartment (**Supplementary Figures S1a, b**).

**Figure 1:**
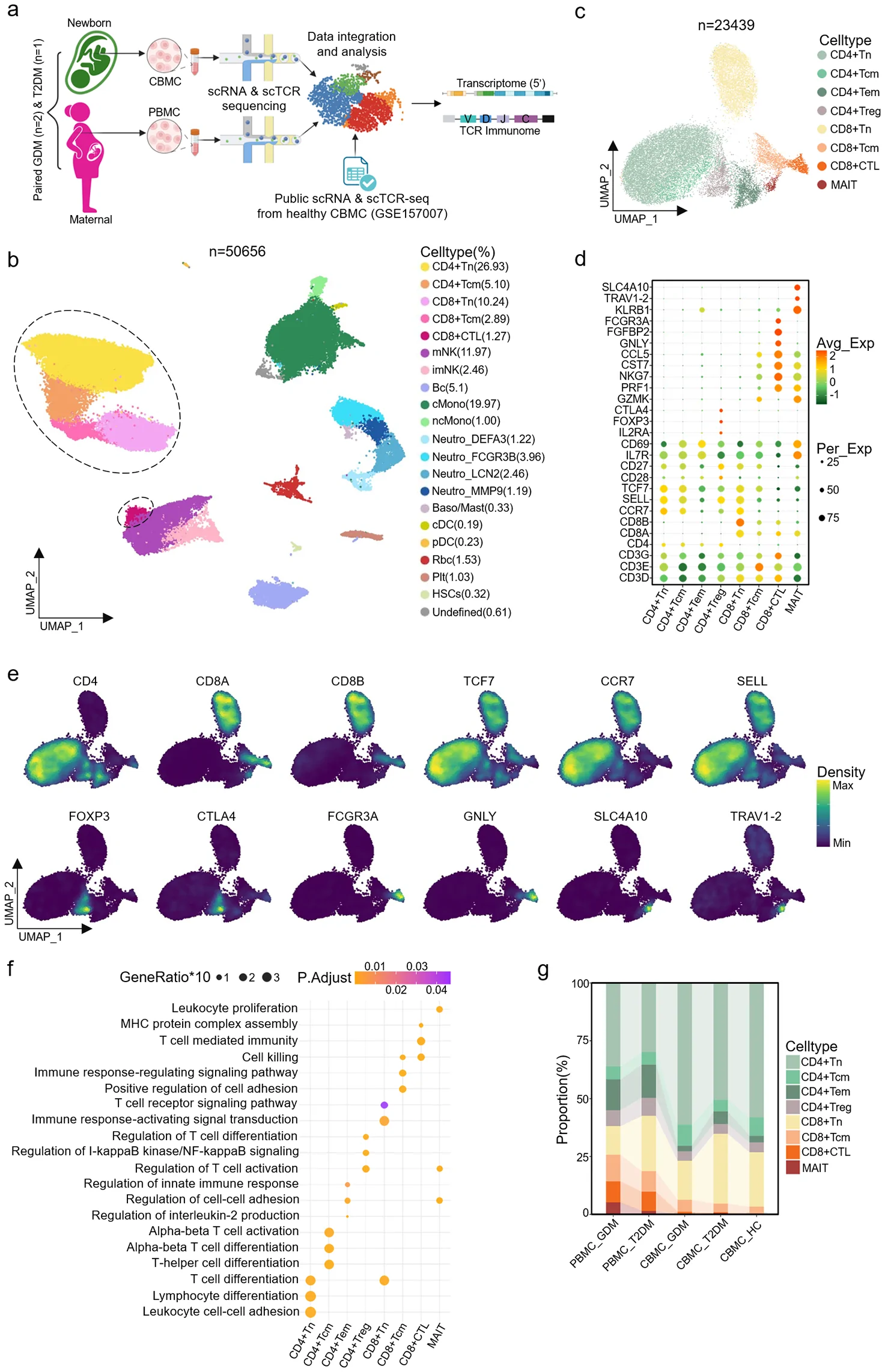
Global single-cell transcriptomic landscape of paired GDM/T2DM mother-neonate samples integrated with healthy cord blood controls. **(a)** Schematic overview of sample collection and the sequencing workflow. Paired PBMC and CBMC samples were collected from GDM/T2DM mothers and their neonates, respectively, and subjected to single-cell RNA sequencing (scRNA-seq) and single-cell TCR sequencing (scTCR-seq). The resulting data were integrated with a public scRNA-seq and scTCR-seq dataset of healthy neonatal cord blood (GSE157007). Following quality control, batch correction, and cell-type annotation, a single-cell transcriptomic atlas of paired maternal-neonatal immune cells was constructed. **(b)** UMAP visualization of all annotated immune cell subsets in the integrated dataset. Each color represents a distinct cell subset, and the total cell number is indicated at the top of each plot. “Undefined” denotes subsets that could not be unambiguously annotated due to the absence of specific marker genes. T-cell subsets are highlighted within black dashed boxes. This labeling convention applies to all UMAP plots in this paper. **(c)** UMAP visualization of T cells from image **(b)** following secondary dimensionality reduction and sub-clustering. **(d)** Dot plot showing the expression of canonical marker genes used to annotate each T-cell subset. Color intensity represents normalized expression level; dot size represents the proportion of cells expressing each gene within the corresponding subset. **(e)** Kernel density plots showing the expression specificity of annotated marker genes across T-cell subsets in UMAP space. **(f)** Dot plot showing the top 3 enriched pathways for each T-cell subset based on cluster-specific differentially expressed genes. Dot size is proportional to gene ratio × 10; color intensity represents the adjusted p value. This labeling convention applies to all functional enrichment dot plots in this paper. **(g)** Stacked bar plot showing the relative abundance of T-cell subsets across sample groups.

After batch correction, our dataset (30,736 cells) and public control dataset (19,920 cells) were integrated into a total of 50,656 high-quality cells for clustering. Consistent cell-type distributions and canonical marker expression validated reliable integration (**Supplementary Figures S1c, d**). Immune subsets were annotated against published human blood immune atlases (6, 7). The major marker genes used for annotation included Tn (TCF7^hi^, CCR7^hi^, SELL^hi^), Tcm (TCF7^+^, CCR7^+^, CD69^hi^), CD4^+^ Treg (FOXP3^hi^, CTLA4^hi^), CD8^+^ CTL (TCF7^-^, CCR7^-^, PRF1^hi^, NKG7^hi^), MAIT cells (TRAV1-2^hi^, SLC4A10^hi^), classical monocytes (CD14^hi^, IL1B^hi^, CXCL8^hi^), non-classical monocytes (FCGR3A^hi^, MS4A7^hi^, CDKN1C^hi^), mature NK cells (FCGR3A^hi^, PRF1^hi^, NKG7^hi^), immature NK cells (NCAM1, XCL1), neutrophil subsets (CD68^-^, DEFA3^hi^, FCGR3B^hi^, LCN2^hi^, MMP9^hi^), B cells (MS4A1^hi^, CD79B^hi^), basophil or mast cells (Baso/Mast, HDC^+^, SLC24A3^+^) plasmacytoid dendritic cells (CLEC4C^hi^, IL3RA^hi^), classical dendritic cells (CD1C^hi^, FCER1A^hi^) and hematopoietic stem cells (HSCs, CD34^hi^, SPINK2^hi^). Our CD45^+^/CD45^−^ enrichment retained more neutrophils than standard separation used in GSE157007 (Materials and Methods), thus all neutrophils were removed to eliminate granulocyte-derived compositional bias (**Supplementary Figure S1c**).

Maternal and neonatal immune profiles were sharply divergent (**Supplementary Figure S1e**). Memory and cytotoxic T cells dominated maternal PBMCs, while naïve CD4^+^ T cells prevailed in cord blood, matching known neonatal immune physiology. Within cord blood, classical monocytes were slightly expanded and naïve CD4^+^ T cells were reduced in hyperglycemia-exposed samples compared to healthy controls. Such lineage skewing lays a cellular foundation for subsequent functional analysis of individual immune populations.

### Increased CD8^+^ central memory T cells in CBMC exposed to intrauterine hyperglycemia

As core adaptive immune regulators, T cells undergo metabolic and functional remodeling under diabetic stress. Intrauterine hyperglycemia can permanently program fetal immune function (2). We extracted 23,439 T cells for high-resolution subclustering and resolved eight distinct subsets (**Figures 1b, c**), each defined by unique canonical markers verified via kernel density plots: CD4^+^/CD8^+^ Tn (TCF7^hi^, CCR7^hi^), CD4^+^/CD8^+^ Tcm (TCF7^+^, CCR7^lo^), CD4^+^ Tem (TCF7^lo^, CCR7^-^), CD4^+^ Treg (FOXP3^hi^, IL2RA^hi^, CTLA4^hi^), CD8^+^ CTL (GNLY^hi^, FGFBP2^hi^, FCGR3A^hi^), MAIT (KLRB1^hi^, TRAV1-2^hi^, SLC4A10^hi^) (**Figures 1d, e**). Functional enrichment matched each subset’s identity: Tn participated in T cell differentiation; CD4^+^ Treg mediated immune suppression; CD8^+^ CTL exerted cytotoxic effects; MAIT cells displayed innate-like MHC-associated activity (**Figure 1f**).

CD4^+^ Tn constituted the major T population across all samples. In comparison to maternal PBMCs, all cord blood samples contained more CD8^+^ Tn and fewer effector/memory T cells. Notably, CBMC_GDM and CBMC_T2DM exhibited moderately elevated CD8^+^ Tcm fractions compared with healthy neonatal cord blood (**Figure 1g**).

### Hyperglycemia reduces neonatal TCR diversity and shortens CDR3

We performed comprehensive TCR clonotype profiling to assess repertoire changes under gestational hyperglycemia. T cells were categorized by clone size into Rare (1 cell), Small (2–10 cells), Medium (11–50 cells), and non-αβ TCR clones. Expanded Small/Medium clones mainly corresponded to CD8^+^ CTL and CD8^+^ Tcm, while rare clones distributed evenly across all T subsets (**Figure 2a**).

**Figure 2:**
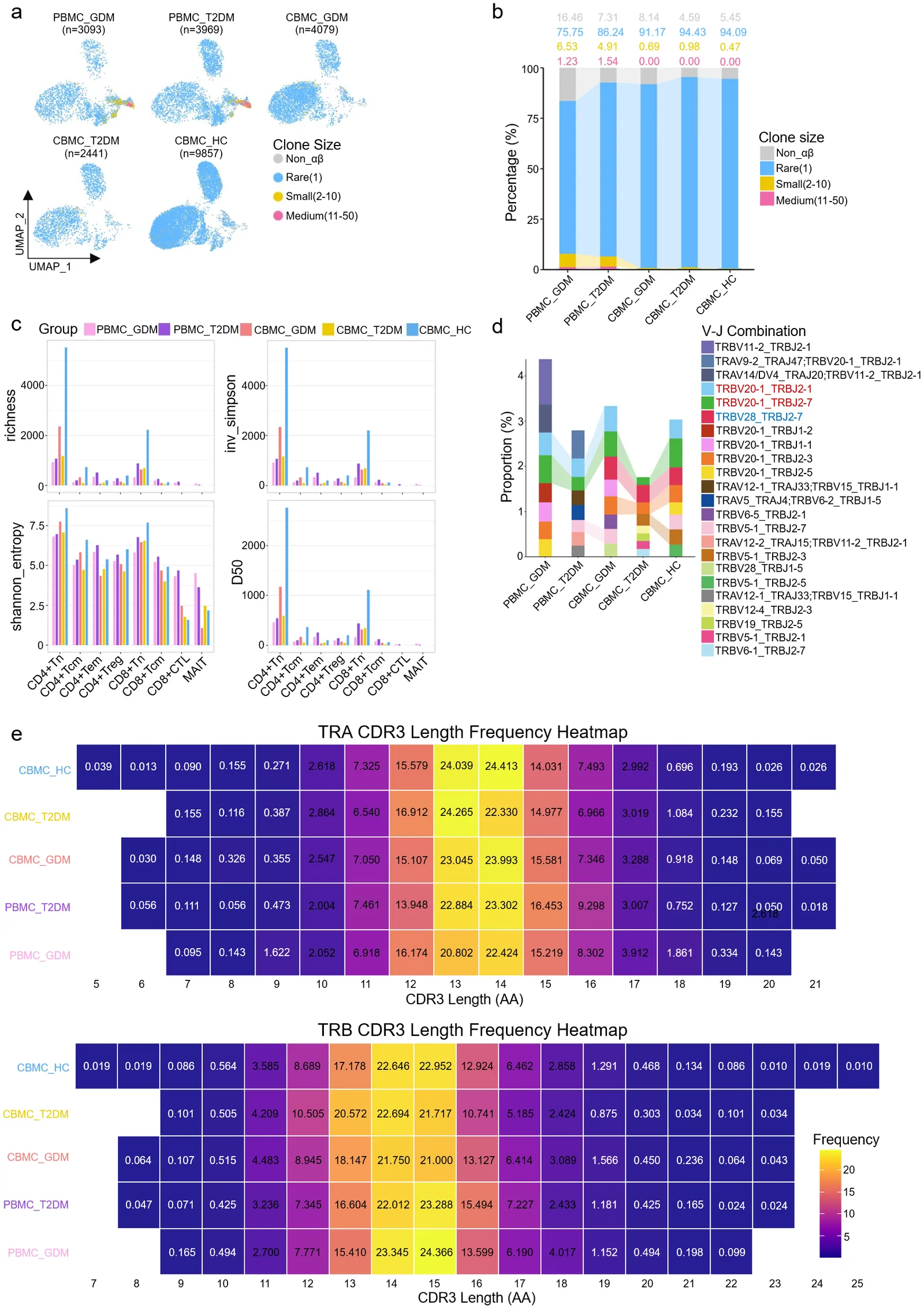
TCR immune repertoire clonal structure and diversity differences across sample groups. **(a)** UMAP visualization showing the distribution of clonotype categories (Rare, Small, Medium, and Non_αβ) across sample groups. **(b)** Stacked bar plot showing the proportional composition of clonotype categories within each sample group. Each bar represents one group, and each color corresponds to a clonotype size category. **(c)** Grouped bar plot showing between-group differences in TCR diversity indices (richness, inverse Simpson index, Shannon entropy, and D50) within each T-cell subset. **(d)** Stacked bar plot showing the proportional representation of the top 8 TRBV-TRBJ gene combinations in each sample group. Each color represents one TRBV-TRBJ combination. **(e)** Heatmap showing the CDR3 amino acid (AA) length usage frequency of TRA (upper panel) and TRB (lower panel) chains across sample groups. Color intensity represents frequency; group labels and CDR3 length positions are indicated on the left and bottom of each panel, respectively. Frequency values below 2% are labeled in white; all others are labeled in black.

Rare clones made up over 91% of cord blood T cells. Adult maternal PBMCs carried more expanded memory clones, whereas cord blood had negligible medium clones; GDM/T2DM cord blood exhibited slightly higher small clone proportions than healthy controls, consistent with the observation that hyperglycemia exposure is not strongly associated with neonatal T cell clonal expansion in this cohort (**Figure 2b**). Four diversity metrics (richness, inverse Simpson, Shannon entropy, D50) showed naive T cells held the highest repertoire diversity, while cytotoxic and MAIT subsets had restricted diversity. All cord blood groups displayed greater overall diversity than maternal PBMCs, yet healthy cord blood possessed marginally higher diversity than hyperglycemia-exposed neonates (**Figure 2c**).

Top frequent V–J pairs including TRBV20-1_TRBJ2–7 existed universally across all groups, unaffected by hyperglycemia. TRBV28_TRBJ2–7 was exclusively enriched in neonatal cord blood as a developmental stage-specific public clonotype (8); TRBV20-1_TRBJ2–1 was also consistently detected, indicating that these V–J combinations represent high-frequency public clonotypes broadly distributed across age groups and not significantly altered by hyperglycemic exposure (**Figure 2d**; **Supplementary Figure S2a**). Only limited shared V–J rearrangements existed within each maternal-neonatal dyad, consistent with placental immune separation and independent fetal TCR repertoire formation (**Supplementary Figure S2b**).

We further analyzed the CDR3 length frequency of the TRA and TRB chains. TRA CDR3 sequences peaked at 14 amino acids in length in all samples, with higher peak frequencies in cord blood. TRB CDR3 peaked at 15 amino acids in length in maternal PBMCs versus 14 amino acids in cord blood. Healthy cord blood covered a wider range of ultra-short and ultra-long TRB CDR3 sequences, while GDM/T2DM cord blood shifted toward shorter CDR3 lengths with narrower distribution (**Figure 2e**). In diabetes, shortened CDR3 sequences and narrowed repertoires are linked to reduced TCR diversity, uneven repertoire distribution and higher autoreactive T cell potential (9). Collectively, shortened TRB CDR3 intervals in GDM cord blood mirror diabetic TCR-repertoire signatures, yet potential links to altered immune recognition or autoimmune susceptibility cannot be resolved by these cross-sectional data and await longitudinal follow-up.

### Hyperglycemia‑associated Tpex‑like T cells in unexpanded neonatal clones

We scored T cell subsets with four functional gene signatures [Inflammation (10), Activation (11), Tolerant (11, 12), and Induced_age (13)] to assess group-specific functional shifts (**Figure 3a**; **Supplementary Table S2**). Relative to healthy cord blood, GDM/T2DM CBMCs displayed elevated Activation and Induced_age scores, while all CBMC groups showed lower Tolerant scores than maternal PBMCs. Stemness genes TCF7, LEF1 (14), and pre-exhaustion transcription factors TOX (15) and IKZF2 (16) were markedly upregulated in CD8^+^ Tn and CD8^+^ Tcm from hyperglycemic neonates (**Figure 3b**). Co-upregulation of TCF7 and TOX indicated fetal precursor T cells acquire characteristic precursor exhausted (Tpex) transcriptional profiles under intrauterine hyperglycemia (14, 17).

**Figure 3:**
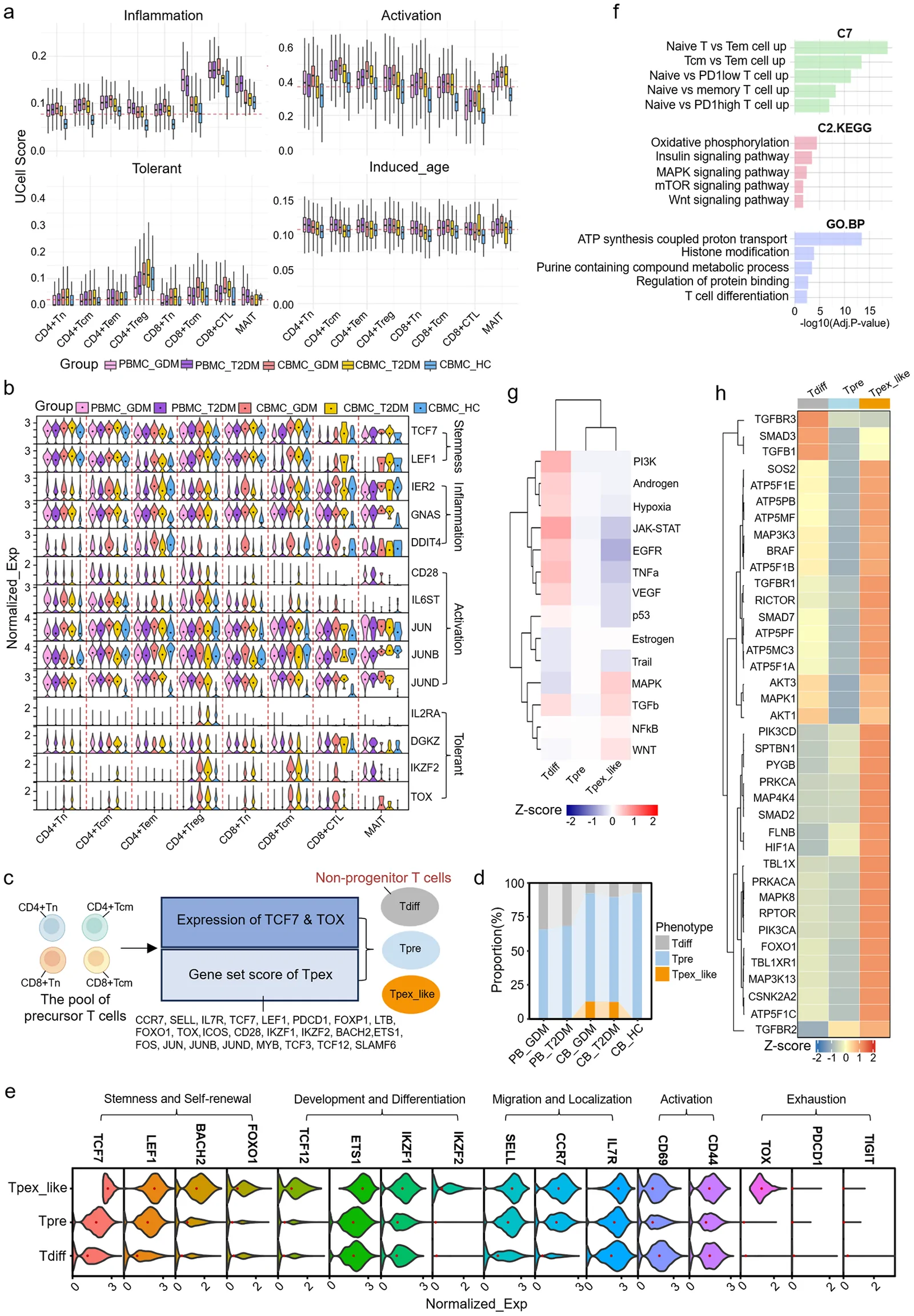
Identification and transcriptional characterization of the Tpex-like phenotype. **(a)** Box plots showing between-group differences in multiple functional gene set scores across T-cell subsets. Each group is represented by a distinct color. The solid black line within each box indicates the median; the red dashed line indicates the overall median of the corresponding gene set. **(b)** Violin plots showing between-group differences in the expression of representative genes from the functional gene sets shown in image **(a)** across T-cell subsets. Black dots within the violin plots indicate the median expression value for each group. **(c)** Schematic diagram illustrating the T-cell phenotype classification framework. Within the precursor T-cell pool (CD4^+^ Tn, CD4^+^ Tcm, CD8^+^ Tn, and CD8^+^ Tcm), cells with a Tpex gene set score above the 75th percentile and with both TCF7 and TOX expression above the 75th percentile are defined as Tpex-like. Remaining precursor T cells are classified as Tpre, and all non-precursor T cells are classified as Tdiff. The genes comprising the Tpex gene set are listed at the bottom of the panel. **(d)** Stacked bar chart illustrating the proportional distribution of phenotypes across experimental sample groups. **(e)** Violin plots comparing the expression levels of canonical Tpex signature markers among phenotypes. Red dots within each violin denote the median expression value. **(f)** Bar plot showing enriched pathways based on genes significantly upregulated in Tpex-like cells relative to other T-cell phenotypes (avg_log2FC > 0.8, adjusted p < 0.05). C7: MSigDB immunologic signature gene sets; C2.KEGG: Kyoto Encyclopedia of Genes and Genomes pathways; GO.BP: Gene Ontology Biological Process terms. **(g)** Heatmap showing pathway activity across T-cell phenotypes. Color intensity represents the corrected pathway activity score (Z-score). **(h)** Heatmap showing the expression of genes from classical signaling pathways across T-cell phenotypes. Color intensity represents the corrected expression level (Z-score). The pathways and corresponding genes displayed are as follows: 1. Oxidative phosphorylation: ATP5F1E, ATP5F1C, ATP5MC3, ATP5F1B, ATP5MF, ATP5F1A, ATP5PB, ATP5PF; 2. TGF-β pathway: TGFB1, TGFBR1, TGFBR2, TGFBR3, SMAD2, SMAD3, SMAD7, SPTBN1; 3. Insulin signaling pathway: PIK3CD, FOXO1, MAPK8, PIK3CA, PRKACA, PYGB, SOS2; 4. MAPK and mTOR signaling pathways (shared genes): MAPK1, MAP4K4, MAPK8, AKT1, AKT3, PRKCA, FLNB, PRKACA, MAP3K13, SOS2, MAP3K3, HIF1A, RICTOR, BRAF, RPTOR; 5. Wnt signaling pathway: MAPK8, TBL1X, PRKCA, PRKACA, TBL1XR1, CSNK2A2.

Drawing on established human T cell classification standards (11), we categorized CD4^+^/CD8^+^ Tn and Tcm cells into three phenotypes (**Figure 3c**): Tpex-like cells (top 25% Tpex signature, high TCF7 and TOX), Tpre (stemness-positive cells failing Tpex thresholds), and differentiated Tdiff cells. We next assessed the relative proportions of Tdiff, Tpre, and Tpex‑like cells across sample groups (**Figure 3d**). Consistent with earlier observations, Tpex‑like cells occupied a measurable fraction of precursor T cells only within hyperglycemic cord‑blood samples, while this population remained negligible in other groups. In addition, most canonical Tpex marker genes exhibited higher median expression within Tpex-like cells relative to both Tpre and Tdiff cells, reinforcing the transcriptional resemblance between our neonatal subset and reported Tpex profiles (41). Specifically, markers sustaining Tpex stemness (TCF7, FOXO1, BACH2), genes supporting early T-cell development (IKZF1, IKZF2), and molecules mediating T-cell homing and migration (SELL, CCR7, IL7R) showed significantly higher expression in Tpex-like cells compared with Tpre and Tdiff cells (42, 43). Markers associated with T-cell activation (CD44, CD69) (44, 45) were expressed at higher levels in Tpex-like cells than in Tpre cells but lower than in Tdiff cells (**Figure 3e**).

Enrichment analyses confirmed unique Tpex-like transcriptional identities (**Figure 3f**). C7 immunologic signatures highlighted robust self-renewal programs matching combined stemness and early exhaustion traits. C2 KEGG pathways enrichment analysis suggested oxidative phosphorylation, insulin, MAPK, mTOR and Wnt signaling, mirroring pathway remodeling in chronically stimulated Tpex cells (18). GO biological process terms further revealed epigenetic and metabolic reprogramming within this subset.

We quantified 17 canonical signaling activities via decoupleR regression (**Figure 3g**). Tpex-like cells had the strongest TGF-β, MAPK and WNT pathway activity, alongside moderate PI3K and JAK-STAT signaling; Tdiff cells featured dominant pro-inflammatory androgen, hypoxia, TNF-α and VEGF pathways. Target gene expression validated these pathway differences: TGF-β receptors, oxidative phosphorylation components, MAPK/mTOR and Wnt mediators were selectively enriched in Tpex-like cells (**Figure 3h**).

In summary, our scoring framework defines a biologically meaningful Tpex-like neonatal T cell phenotype. Its dual stemness/pre-exhaustion signature aligns with canonical Tpex molecular hallmarks (17), and its multi-signaling activation profile matches the Tpex regulatory network documented by Zhou et al. (19). These findings suggest that intrauterine hyperglycemic exposure may be associated with metabolic and signaling alterations in fetal precursor T cells, though direct functional validation is required.

We next mapped the distribution of Tpex-like, Tpre and Tdiff cells across cohorts and clonal groups (**Figure 4**). Tpex-like cells were sharply enriched in Rare unexpanded clones of hyperglycemic cord blood (CBMC_GDM: 12.8%; CBMC_T2DM: 12.32%) but barely detected in small/medium expanded clones, while healthy cord blood contained only 0.39% Tpex-like cells. Maternal PBMCs held negligible Tpex-like populations, and expanded adult clones were overwhelmingly composed of differentiated Tdiff cells. Overall, Tpex‑like cells were selectively enriched within non‑expanded neonatal precursor T clones from hyperglycemia‑exposed cord blood, whereas healthy cord blood is dominated by Tpre cells. This distribution pattern is consistent with an association between gestational hyperglycemic exposure and altered neonatal T‑cell self‑renewal‑ and metabolic‑related transcriptional programs (17).

**Figure 4:**
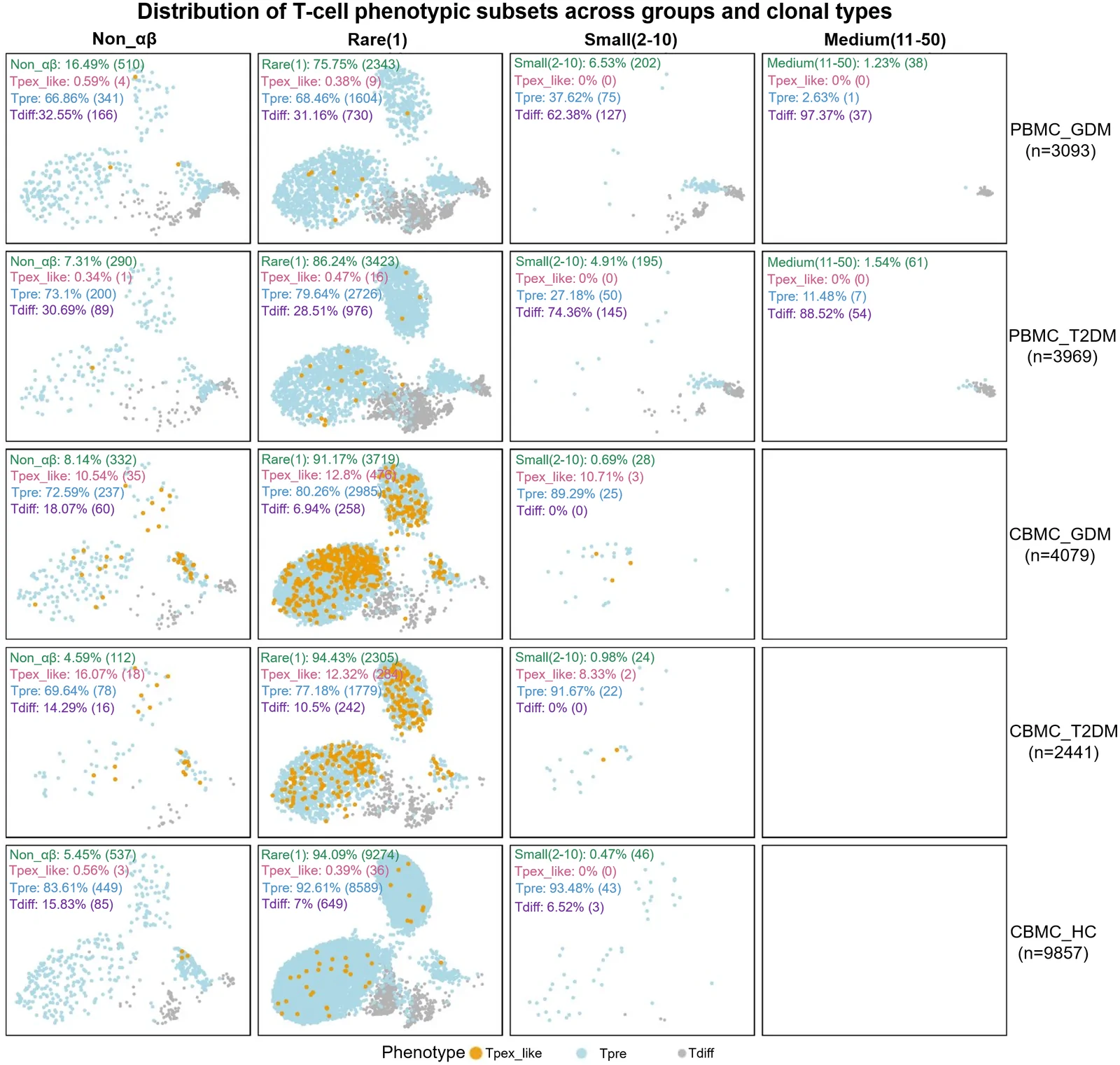
Distribution of T-cell phenotypes across sample groups and clonotype categories. Faceted UMAP plots showing the abundance and distribution of T-cell phenotypes within each clonotype category (Rare, Small, Medium, and Non_αβ) across sample groups. Group labels are indicated on the right; the total number of T cells in each group is shown in parentheses. Tpex-like, Tpre, and Tdiff cells are represented by pink, blue, and purple dots, respectively. The proportion and absolute number of each phenotype within each clonotype category and the Non_αβ fraction are annotated in green.

SCENIC analysis was applied to uncover upstream transcription factors governing the Tpex-like state, screening active regulons with Z-scores above 1. Overlapping active regulons from Tpex-like cells, GDM cord blood and TOX modulators pinpointed two core transcription factors: TCF7 and FOXP1 (**Supplementary Figure S3a**). Both exhibited elevated activity in Tpex-like cells and robust links to TOX-associated regulatory programs.

We characterized significantly upregulated target genes within their respective regulons (**Supplementary Figure S3**). The TCF7 regulon covers stemness and exhaustion markers including LEF1, TOX and FOXO1; FOXP1 shares partial targets such as IKZF1, IKZF2 and LEF1, many of which are canonical Tpex signature genes. Joint control of stemness and pre-exhaustion programs by TCF7 and FOXP1 recapitulates known Tpex regulatory architectures. Single-cell CRISPR work has validated IKAROS and ETS1 as critical factors restraining Tpex terminal differentiation, with ETS1 loss triggering hyperactive mTORC1 metabolism (19). Concurrent activation of TCF7, LEF1 and ETS1 in our dataset sustains Tpex self-renewal and blocks full exhaustion, revealing this dual TF network as the molecular basis for Tpex-like accumulation in hyperglycemic neonatal cord blood.

In pseudotime space, Tpre and Tpex‑like cells occupied early‑to‑middle pseudotime segments with partial spatial overlap, while Tdiff cells predominantly resided within late‑pseudotime terminal regions (**Supplementary Figure S3c**). We assessed the distribution of Tpex‑associated gene‑set signature scores (42) across pseudotime, separated by cellular phenotype. Tpex‑like cells exhibited the highest Tpex‑gene‑set signature activity along trajectories, followed by Tpre cells, whereas Tdiff cells displayed the lowest signature magnitude (**Supplementary Figure S3c**). We next examined dynamic gene‑expression patterns for key regulators of T‑cell stemness and exhaustion (**Supplementary Figure S3d**). Stem-associated genes TCF7, BACH2 (46), FOXO1 (47) and IKZF1 (48) gradually declined over pseudotime. In contrast, TOX and IKZF2 reached peak activity within the Tpex‑like compartment, decreased among Tpre cells, and were re‑elevated in terminal Tdiff cells, consistent with published functional descriptions of these exhaustion-related transcription factors (15, 49). Collectively, these multi‑layer pseudotime‑associated observations further support Tpex‑like cells as a distinct intermediate transcriptional state rather than a simple admixture of Tpre and terminally‑differentiated Tdiff populations.

### Hyperglycemia expands pro-inflammatory classical monocytes in neonatal innate immunity

We isolated 19,688 innate immune cells (monocytes, NK cells, B cells) for subclustering to support subsequent cell-cell crosstalk analysis, resolving 14 distinct subsets (**Supplementary Figure S4a**). We annotated classical monocytes (cMono, CD68^hi^, CD14^hi^, LYZ^hi^) and three functionally specialized cMono variants: cMono_LGALS2 (LGALS2^hi^), cMono_ISGs (MX1^hi^, ISG15^hi^), and cMono_CXCL8 (CXCL8^hi^, IL1B^hi^); non-classical monocytes (ncMono, FCGR3A^hi^, MS4A7^hi^); monocyte-platelet aggregates (MPAs, PPBP^hi^, PF4^hi^); and neutrophil-like monocytes (neuMono, LTF^hi^, LCN2^hi^, PGLYRP1^hi^). NK cell subsets comprised mNK_KLRC2 (KLRC2^hi^, NKG7^hi^), mNK_CD160 (CD160^hi^, KLRB1^hi^), intermediate NK cells (intNK, intermediate GZMB and NCAM1 expression), and immature NK cells (imNK, NCAM1^hi^, XCL1^hi^). B cell subsets included transitional B cells (transB, TCL1A^hi^), naive B cells (nB, MS4A1^hi^, IGHD^hi^), and memory B cells (mB, CD27^hi^, AIM2^hi^, IGHA1^hi^). All subsets were validated through lineage-specific marker gene expression (**Supplementary Figure S4b**), and the compositional proportions and marker expression patterns of monocyte subsets were consistent with published GDM cord blood myeloid cell atlases (20).

Cluster-specific DEG-based GO enrichment validated subset functional identities (**Supplementary Figure S4c**): monocytes dominated innate immune and cytokine regulatory pathways, NK cells mediated cytotoxic programs, and B cells featured BCR signaling and differentiation signatures. Cross-group proportion comparisons showed expanded pro-inflammatory cMono_ISGs and cMono_CXCL8 in maternal hyperglycemic PBMCs, and elevated total classical monocytes in GDM/T2DM cord blood. mNK_KLRC2 and naive B cells also displayed divergent frequencies between hyperglycemic and healthy neonatal groups (**Supplementary Figure S4d**), laying a cellular foundation for intercellular communication exploration.

### Pro‑inflammatory and homeostatic immune‑crosstalk signatures in hyperglycemia‑exposed neonatal cord blood

We used CellChat to compare immune intercellular signaling between CBMC_GDM and CBMC_HC and explore hyperglycemic network remodeling. GDM cord blood exhibited more numerous and stronger immune cell interactions (**Figure 5a**). Pro-inflammatory axes SEMA4, ADGRE, IL-16, SIRP and PECAM2 prevailed in CBMC_GDM (21–23), while immune-homeostatic LAIR1 (24), GALECTIN (25) and GRN (26) pathways dominated healthy controls. These predicted differences suggest that pro-inflammatory ligand-receptor interactions may be enriched and homeostatic pathways relatively attenuated in hyperglycemia-exposed neonatal cord blood.

**Figure 5:**
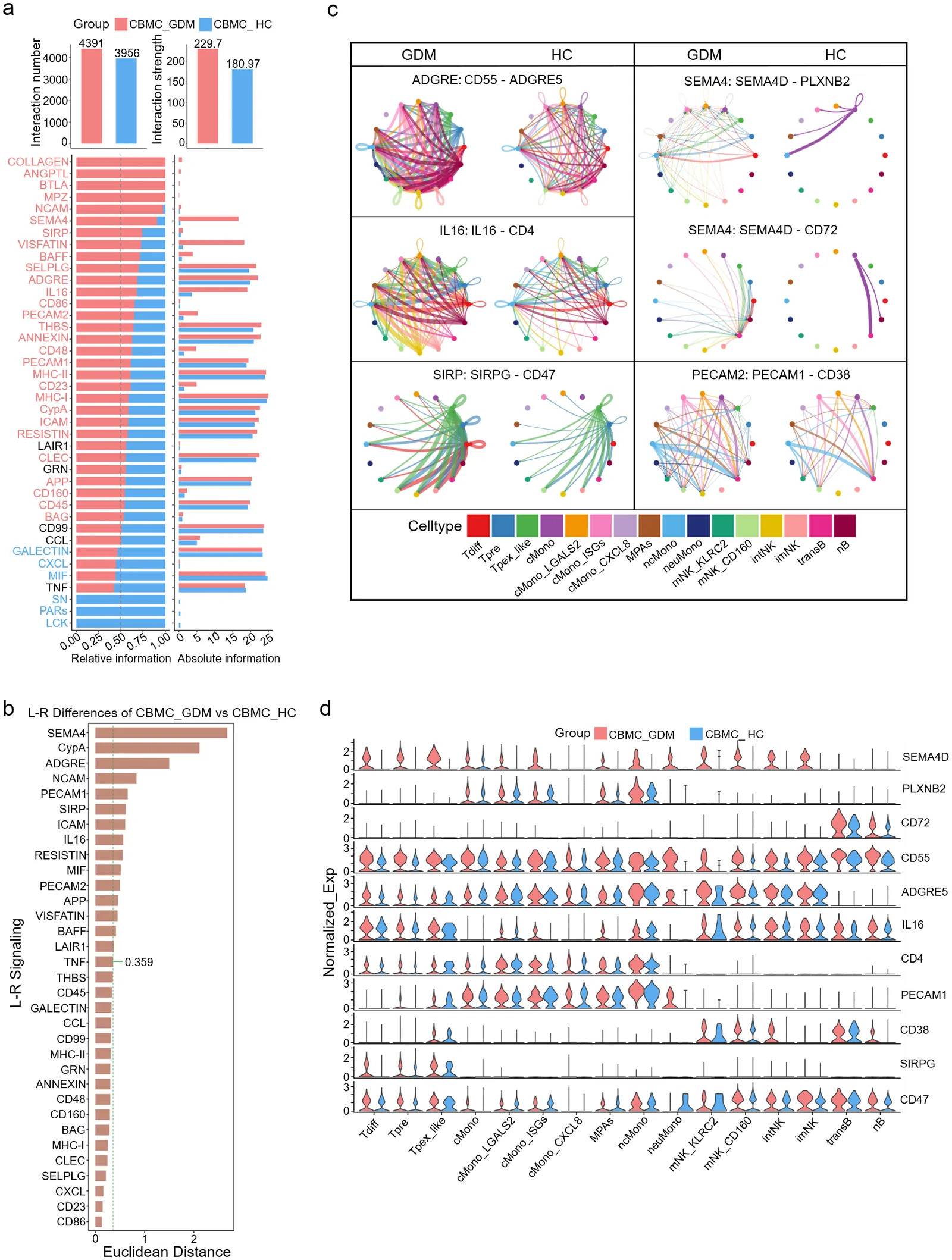
Differences in cell-cell communication between T-cell phenotypes and monocytes, NK cells, and B cells in CBMC_GDM versus CBMC_HC. **(a)** Upper panel: bar plots comparing the total number of inferred cell-cell interactions and the overall interaction strength between CBMC_GDM (red) and CBMC_HC (blue). Lower-left panel: horizontal stacked bar plot showing the relative signaling intensity of multiple ligand-receptor (L-R) pathways in each group. Lower-right panel: grouped bar plot showing absolute signaling strength differences across L-R pathways between the two groups. L-R pathways significantly enriched in CBMC_GDM are highlighted in red; those enriched in CBMC_HC are highlighted in blue. **(b)** Bar plot showing the Euclidean distance of information features for each L-R signaling pathway between CBMC_GDM and CBMC_HC. The green dashed line indicates the median Euclidean distance (0.359), which was used as a cutoff to identify pathways with the most significant differences. **(c)** Circle plots illustrating the differences in cell-cell communication strength across differentially active L-R signaling pathways. The ligand-receptor pair of each pathway is indicated in the panel title. Each color represents one cell type; arcs originate from the sender cell type, matching its color, and point toward the receiver cell type. Arc width is proportional to signal strength. **(d)** Violin plots showing between-group differences in the expression of ligand and receptor genes from the L-R signaling pathways shown in image **(c)**. The name of each corresponding L-R pathway is indicated on the right.

We calculated Euclidean distance to screen divergent ligand-receptor axes with a median cutoff of 0.359 and analyzed eight top differential pathways (**Figures 5b, c**). The SEMA4D–PLXNB2/CD72 axis driving T cell and myeloid inflammation was highly enriched in GDM samples (21, 22). CD55–ADGRE5 formed extensive connections across T cells, monocytes and NK cells only in CBMC_GDM. IL-16–CD4, which recruits and activates CD4^+^ T cells, was also upregulated in hyperglycemic cord blood (23). Predicted higher SIRPG‑CD47 signaling is consistent with previously‑described patterns of disrupted innate‑adaptive immune recognition (27), while elevated PECAM1–CD38 facilitated monocyte adhesion and activation (28, 29).

Violin plots verified subset-specific ligand-receptor expression consistent with network results (**Figure 5d**). SEMA4D, IL-16 and SIRPG were concentrated in GDM T cells; CD55 and PECAM1 were abundant in GDM monocytes; corresponding receptors were mostly restricted to innate/B cell subsets in healthy cord blood.

In summary, hyperglycemic exposure is associated with predicted remodeling of neonatal immune communication toward a pro‑inflammatory pattern, with amplified inflammatory ligand‑receptor crosstalk and diminished homeostatic signaling. These inter‑cellular network observations provide transcriptome‑derived computational evidence for potential immune‑signaling alterations and warrant further functional validation.

## Discussion

To understand how intrauterine hyperglycemic exposure shapes neonatal T cell lineage development and TCR repertoire architecture in GDM, in this exploratory descriptive single-cell multi-omic study, we firstly identified candidate multi-layered neonatal immune alterations associated with GDM-related intrauterine hyperglycemia exposure. At the cellular composition level, neonates born to GDM and T2DM mothers displayed expanded classical monocytes and reduced naive T cell frequencies relative to healthy cord blood controls, representing a shared myeloid-biased developmental shift. This aligns with Yin et al. (20), who detected CXCL8^+^IL1B^+^ monocyte expansion in GDM cord blood, and the broader model that hyperglycemia prioritizes myeloid inflammatory programming (30). Diminished naive T cell pools may stem from disrupted thymic signaling or lymphoid progenitor development, supporting a systematic review by Yan et al. (2), that summarized GDM-linked fetal immune dysfunction. Consistently, hematopoietic stem cell homeostasis is highly sensitive to metabolic cues. GDF15-mediated stem cell remodeling further supports that gestational metabolic stress durably reshapes hematopoietic output and immune subset ratios during critical developmental windows (38).

At the TCR repertoire level, hyperglycemic cord blood carried moderately reduced clonal diversity, mirroring restricted TCR diversity seen in adult diabetic patients (31). Since neonatal TCR‑repertoire diversity contributes to lifelong pathogen‑defense capacity, early‑life TCR restriction may represent a candidate marker of immune vulnerability, though long‑term immune risks cannot be determined from our cross‑sectional observations. TRB CDR3 sequences were shorter, with fewer ultra‑long variants, in cord‑blood samples exposed to intrauterine hyperglycemia, this structural signature has been reported in adult autoimmune and diabetic contexts (9), and its potential functional significance in neonates remains to be explored through long‑term prospective follow‑up. Ubiquitous public V–J pairs TRBV20-1_TRBJ2–7 and TRBV20-1_TRBJ2–1 appeared across all maternal and neonatal groups, consistent with conserved high-frequency neonatal clonotypes unaffected by maternal hyperglycemia. By contrast, TRBV28_TRBJ2–7 was cord blood-specific (8, 32), reflecting fetal-stage biased TCR recombination driven by low TdT activity during gestation (33). Collectively, this exploratory study provides the first single-cell level evidence in GDM-exposed neonatal cord blood linking intrauterine hyperglycemia to a composite TCR phenotype — moderately compressed diversity, shortened CDR3 intervals, and preserved overall evenness within the rare clonotype compartment that harbors the Tpex-like subset described below. These correlative observations may serve as candidate molecular clues for future investigations into how gestational metabolic stress shapes neonatal TCR architecture and lineage characteristics, and await longitudinal validation.

We identified a unique Tpex-like subset selectively enriched in hyperglycemic neonatal cord blood, co-expressing stemness (TCF7, LEF1) and pre-exhaustion (TOX, IKZF2) markers. Transcriptional comparison across the three precursor phenotypes further delineated a gradient of differentiation-associated markers that distinguishes Tpex-like cells from Tpre and Tdiff populations (**Figure 3e**). Specifically, Tpex-like cells expressed the highest levels of stemness-sustaining factors TCF7 and BACH2 — the latter of which has been shown to restrain effector differentiation by competing with AP-1 factors at effector-gene enhancers, thereby preserving quiescence and self-renewal capacity in both memory and Tpex contexts (50). Concurrently, Tpex-like cells displayed elevated IKZF1 and IKZF2 relative to Tpre cells, consistent with a transcriptional profile reminiscent of earlier lymphoid developmental stages, as Ikaros-family factors are known to regulate lymphocyte lineage commitment and early T-cell progenitor maintenance (51). Intriguingly, the activation markers CD44 and CD69 were expressed at intermediate levels in Tpex-like cells — higher than Tpre but lower than Tdiff — suggesting that this subset occupies a state of early activation without terminal commitment, analogous to the “sensing” phenotype recently described in progenitor-exhausted T cells under chronic antigen exposure (44, 45). Canonical Tpex populations in tumors and chronic infection rely on sustained antigen stimulation to sustain self-renewal and replenish exhausted T cells (17), with TOX induction driven by calcineurin–NFAT2 signaling under persistent TCR triggering (34). However, neonatal Tpex-like cells resided exclusively within unexpanded rare clonotypes without antigen-driven clonal expansion, suggesting that intrauterine hyperglycemia may initiate this dual stemness/exhaustion transcriptional state potentially via metabolic and epigenetic reprogramming.

Functional pathway profiling revealed coordinated activation of mTOR, TGF-β and WNT signaling, matching known Tpex regulatory signatures. Single-cell CRISPR data from Zhou et al. (19) established mTOR as a core Tpex fate controller, while TGF-β preserves Tpex mitochondrial integrity and stemness by restraining mTORC1 activity (18). Our data suggest synergistic mTOR/TGF-β signaling is associated with the neonatal Tpex-like phenotype, though direct functional validation is still needed. Elevated WNT signaling further sustains TCF7 expression to maintain stemness and avoid terminal exhaustion (14, 17). SCENIC regulon analysis pinpointed TCF7 and FOXP1 as master co-regulators of this subset. FOXP1 preserves naive T cell quiescence (35) and suppresses PI3K–AKT–mTOR metabolism (36), and overlapping target genes (LEF1, IL7R) enable coordinated transcriptional programming toward the Tpex-like state. Conserved regulon activity of IKZF1, IKZF2 and ETS1 recapitulates the differentiation control network reported by Zhou et al. (19). Enrichment of histone modification pathways in Tpex-like cells additionally confirms hyperglycemia pre-establishes exhaustion-associated epigenetic landscapes independent of chronic antigen exposure (34). The exclusive localization of Tpex-like cells within non-expanded clones is consistent with the hypothesis that this state is not primarily driven by antigen-driven clonal expansion, raising the possibility of cell-intrinsic transcriptional alterations associated with intrauterine metabolic conditions.

Pseudotime trajectory analysis further positioned Tpex-like cells at an intermediate differentiation state between Tpre and Tdiff cells along the precursor-to-effector axis (**Supplementary Figures S3c, d**). Along pseudotime, stemness-associated factors (TCF7, BACH2, FOXO1, IKZF1) declined progressively, whereas the exhaustion-related regulators TOX and IKZF2 peaked specifically within the Tpex-like compartment. These dynamic expression patterns suggest that Tpex-like cells may simultaneously maintain self-renewal capacity while pre-establishing exhaustion-associated epigenetic programs (15, 34, 50). CellChat intercellular communication analysis predicted a globally altered immune signaling network in GDM cord blood, with greater total interaction counts and signal strength than healthy controls. Pro-inflammatory axes including SEMA4, IL-16, ADGRE and SIRP were amplified in hyperglycemic neonates, whereas homeostatic inhibitory pathways (LAIR1, GALECTIN, GRN) dominated healthy cord blood, implying homeostatic inhibitory pathways may be relatively attenuated in hyperglycemia-exposed neonatal cord blood. The SEMA4D–PLXNB2/CD72 ligand-receptor pair was markedly enriched in GDM cord blood; SEMA4D boosts T cell and monocyte inflammatory responses (21) and is implicated in chronic inflammatory disorders (22). Consistent with metabolic inflammation findings from Reyes-Farias et al. (23), IL-16 signaling via CD4 was upregulated in GDM T cells, positioning neonatal precursor T cells as active signal transmitters shaping myeloid function rather than passive responders. Combined with myeloid skewing, compressed TCR diversity and Tpex-like cell accumulation, these hyperactivated inflammatory intercellular signaling pathways together illustrate a comprehensive spectrum of fetal immune developmental alterations associated with intrauterine hyperglycemia. It is important to emphasize that both the pseudotime trajectory and CellChat-derived signaling network should be interpreted as predictive, exploratory inferences rather than direct measurements, requiring orthogonal validation (52, 53). Future *in vivo* GDM animal models — in which gestational metabolic conditions can be temporally manipulated — will be essential for longitudinal tracking of Tpex-like cell emergence, proportions, and functional fate, and for determining whether Tpex-like cells represent a transient, reversible state or a durable priming event with long-term immunological consequences.

Epidemiological data indicate that GDM-exposed offspring face elevated risks of allergic disease, infection susceptibility, and potentially autoimmune conditions in later life (2). The immune alterations described here—moderately compressed TCR diversity and evenness, Tpex-like precursor enrichment within rare clonotypes, and pro-inflammatory shifts in intercellular communication—could collectively contribute to altered immune competence if these transcriptional states persist or influence downstream immune maturation. The Tpex-like transcriptional signature, detectable through surface markers such as TCF7, TOX and PD-1 by flow cytometry, represents a candidate cellular indicator for prospective immune monitoring in GDM-exposed neonates, pending validation in larger cohorts.

Taken together, this exploratory single-cell multi-omic analysis provides the first integrated evidence in GDM-exposed neonatal cord blood of a composite immune phenotype encompassing TCR repertoire alterations and a rare-clonotype-restricted Tpex-like transcriptional state. These correlative observations are not intended to establish causal relationships but rather to generate testable hypotheses and candidate molecular markers that may provide clues for future mechanistic investigations into how intrauterine hyperglycemia shapes lifelong immune susceptibility in offspring.

### Limitations

First, this observational exploratory study has limited statistical power (GDM: n=2 dyads; T2DM: n=1 dyad; controls: n=3); all findings are hypothesis-generating and require validation in larger maternal–neonate cohorts and GDM animal models. Second, despite methodological differences between in-house CD45^+^/CD45^−^ co-enrichment and routine control processing, Harmony integration showed no residual batch effects across non-granulocyte lineages. Third, the Tpex-like classification is transcription-based only; functional validation (proliferation, differentiation, surface markers) is needed. Fourth, pseudotime inference represents a single-snapshot computational reconstruction and requires dynamic sampling in GDM animal models to verify whether Tpex-like cells are induced by intrauterine hyperglycemia. Fifth, cell-cell communication predictions require protein-level and functional confirmation.

## Conclusions

Our exploratory multi-omic single-cell profiling describes a spectrum of fetal immune transcriptional alterations associated with intrauterine hyperglycemia in GDM-exposed neonates, including myeloid skewing, moderately compressed TCR repertoire diversity, pro-inflammatory ligand-receptor communication shifts, and a rare Tpex-like precursor T cell subset enriched within unexpanded clonotypes with correlated TCF7/FOXP1 regulon activity. These transcriptional signatures exhibit pre-exhaustion-like features in neonatal T cells, which raises a correlative hypothesis linking GDM intrauterine exposure to altered lifelong immune susceptibility and provides candidate molecular markers for future mechanistic and translational research. Several inherent limitations — including the small cohort size, cross-sectional design, and limited statistical power — constrain causal interpretation of the cross-sectional small-cohort data. Larger matched maternal-neonatal cohorts, *in vitro* cellular hyperglycemic models and *in vivo* GDM animal models are required to validate these candidate signatures and dissect potential mechanistic pathways. Longitudinal clinical surveillance will further clarify whether the Tpex-like transcriptional signature is associated with sustained T cell alterations in childhood and adulthood. In aggregate, this single-cell atlas delineates candidate immune transcriptional perturbations associated with gestational hyperglycemia and proposes exploratory hypotheses linking metabolic intrauterine stress to fetal immune development, laying a resource base for future translational validation in GDM-exposed neonates.

## Materials and methods

### Study cohorts, ethical approval and mononuclear cell preparation

This research enrolled three mother-neonate dyads: two GDM pairs and one T2DM pair, generating matched maternal PBMC (n=3) and neonatal CBMC (n=3) specimens. Three de-identified healthy cord blood datasets (GSE157007) from GEO served as external control samples, requiring no extra ethical clearance.

Sample acquisition was authorized by the Medical Ethics Committee of Guangzhou Panyu Maternal and Child Health Hospital (Approval No. 2023070481, August 4, 2023). All procedures complied with the Declaration of Helsinki, CIOMS guidelines and China’s biomedical research ethics regulations; all mothers provided signed informed consent before sampling.

Our PBMC and CBMC samples adopted CD45^+^/CD45^−^ co-enrichment to preserve granulocytes, while public control samples (GSE157007) used routine centrifugation. To eliminate processing-induced compositional bias, neutrophils were excluded from all subset proportion analyses. Peripheral blood and cord blood samples were collected for PBMC and CBMC isolation for subsequent single-cell sequencing analyses.

### Single-cell RNA and TCR library construction and sequencing

Cell viability was assessed via trypan blue staining, and samples with viability above 85% were used for library preparation. Single-cell transcriptomic and paired TCR V(D)J enrichment libraries were constructed using the 10x Genomics Next GEM 5′ reagent kit. Following GEM formation, reverse transcription, magnetic bead purification and PCR amplification were performed. Qualified libraries were subjected to paired-end sequencing on the Illumina NovaSeq X Plus platform.

### Raw data processing and gene expression quantification

Raw sequencing data from in-house GDM/T2DM samples and the public GSE157007 dataset were uniformly processed with Cell Ranger (v 7.1.0) based on the GRCh38 reference genome. Low-quality barcodes and UMIs were filtered, and only uniquely mapped exonic reads were retained. Valid cell barcodes were identified using EmptyDrops, and standardized cell-by-gene expression matrices were generated for subsequent analysis.

### Quality control, normalization and batch correction

All expression matrices were analyzed in Seurat (v 4.3.0). Cells were filtered by gene count, UMI number and mitochondrial gene ratio, while DoubletFinder was applied to eliminate doublet cells. Non-coding and pseudogene transcripts were removed to reduce background noise. After LogNormalize normalization, 3000 highly variable genes were selected, followed by PCA analysis. Batch effects across datasets were corrected via Harmony (v 1.2.0) integration. UMAP visualization and Louvain clustering were performed with fixed parameters, and T cells and innate immune cell populations were independently extracted for secondary subclustering.

### Cell-type annotation

Immune cell subsets were manually annotated by integrating published human peripheral blood immune atlases and GDM single-cell research findings, relying on canonical subset marker genes. Annotation reliability was further validated by marker expression patterns and functional enrichment results.

### TCR repertoire analysis

High-confidence productive TCR contigs were processed using the Cell Ranger VDJ pipeline and integrated into Seurat objects. The scRepertoire package was used for downstream repertoire profiling. T cells were categorized into Rare, Small and Medium clonotypes according to CDR3 sequence duplication levels. Multiple diversity indices, V–J gene usage patterns and CDR3 length distributions were systematically compared across sample groups.

### Differential expression analysis and functional enrichment

Cluster-specific differentially expressed genes were identified via Wilcoxon rank-sum test in Seurat with strict screening thresholds. GO, KEGG and MSigDB signature enrichment analyses were performed using hypergeometric tests, with FDR < 0.05 defined as significant. The GSVA algorithm was applied to calculate single-cell pathway activity scores for functional comparison across cell subsets.

### T-cell functional scoring and identification of the Tpex-like phenotype

Four T-cell functional modules, including Inflammation, Activation, Tolerant, and Induced_age, were scored at single-cell resolution using the UCell::AddModuleScore (v 2.0.0) function in Seurat based on published gene sets. Based on reported Tpex signatures (17, 39), precursor T cells (CD4^+^/CD8^+^ Tn/Tcm) were classified into three phenotypes: Tpex-like cells, defined by a Tpex gene-set score above the 75th percentile and both TCF7 and TOX expression above the 75th percentile; Tpre cells with stemness features but no Tpex traits; and differentiated Tdiff cells without precursor characteristics.

### Signaling pathway activity inference

The decoupleR (v 2.0.1) package with a multivariate linear model was utilized to calculate Z-scores of 17 canonical PROGENy signaling pathways. Quantitative pathway activity profiles were generated to compare signaling differences among Tpex-like, Tpre and Tdiff cell phenotypes.

### Transcriptional regulatory network inference

SCENIC (v 1.3.1) was employed to construct single-cell transcriptional regulatory networks, including co-expression network modeling, motif enrichment analysis and AUCell regulon activity quantification. Transcription factors with high regulon activity (Z-score > 1) were screened, and core TCF7 and FOXP1 regulators governing the neonatal Tpex-like phenotype were identified via multi-group intersection analysis.

### Pseudotime trajectory analysis

Pseudotime trajectory inference of precursor T‑cell subsets (CD4^+^/CD8^+^ Tn and Tcm) was performed using Monocle 2 (54) (v 2.22.0). Only precursor T‑cells derived from hyperglycemic cord‑blood samples (CBMC_GDM and CBMC_T2DM) were included for trajectory reconstruction. Healthy cord‑blood and maternal PBMC cells were excluded given the scarcity of Tpex‑like cells in healthy cord blood and immunological independence between maternal and neonatal T‑cell compartments. We evaluated pseudotime‑associated distribution of the published Tpex‑related gene‑set signature (42) and analyzed pseudotime‑dependent expression dynamics of selected stemness‑ and exhaustion‑associated genes. All pseudotime outputs are interpreted as exploratory transcriptome‑level observations.

### Cell-cell communication analysis

CellChat (v 2.1.2) was used to construct and compare immune intercellular signaling networks between GDM-exposed and healthy neonatal CBMCs. Global interaction abundance and strength were quantified, and divergent signaling pathways were screened based on Euclidean distance of signaling features. Key differential ligand-receptor pairs were validated via subset-specific expression visualization.

### Statistical analysis and visualization

All statistical analyses were performed in R (v 4.1.0). Two-group and multi-group comparisons were conducted using Wilcoxon rank-sum test and Kruskal-Wallis test, respectively. Pearson correlation analysis was applied for correlation assessment. FDR-adjusted P < 0.05 indicated statistical significance. A series of professional R packages were used for data processing and graphical visualization.

For all inferential group comparisons—including cellular subset proportions, TCR diversity indices, gene set scores, and pathway activities—values were first aggregated at the donor/sample level. Statistical tests were applied based on the number of biological replicates (n = donors), not the number of individual cells, to avoid pseudoreplication. Cell-level analyses (e.g., DEG identification) are used for annotation and pathway characterization only. Given the small sample sizes (n = 2–3 per group), formal mixed-effects models were not applied; this is acknowledged as a limitation.

## Data Availability

The data presented in this study are deposited in the National Genomics Data Center Genome Sequence Archive (GSA), accession number HRA019438 (https://ngdc.cncb.ac.cn/gsa-human/). The public cord blood scRNA-seq and TCR repertoire dataset used as healthy controls (GSE157007) is openly available through the NCBI Gene Expression Omnibus (https://www.ncbi.nlm.nih.gov/geo/query/acc.cgi?acc=GSE157007).

## Glossary

AA: Amino acid
ADGRE: Adhesion G protein-coupled receptor E
AGEs: Advanced glycation end products
aTF: Active transcription factor
BCR: B cell receptor
CBMC: Cord blood mononuclear cell
CDR3: Complementarity-determining region 3
cMono: Classical monocyte
CTL: Cytotoxic T lymphocyte
DEG: Differentially expressed gene
FDR: False discovery rate
GDM: Gestational diabetes mellitus
GEM: Gel beads-in-emulsion
GEO: Gene Expression Omnibus
GO BP: Gene Ontology biological process
GRN: Progranulin
GSVA: Gene set variation analysis
IADPSG: International Association of Diabetes and Pregnancy Study Groups
IL: Interleukin
imNK: Immature natural killer cell
intNK: Intermediate natural killer cell
ISG: Interferon-stimulated gene
ITIM: Immunoreceptor tyrosine-based inhibitory motif
KEGG: Kyoto Encyclopedia of Genes and Genomes
L-R: Ligand-receptor
MAIT: Mucosal-associated invariant T cell
mB: Memory B cell
MLM: Multivariate linear model
mNK: Mature natural killer cell
MPA: Monocyte-platelet aggregate
mTOR: Mammalian target of rapamycin
nB: Naïve B cell
ncMono: Non-classical monocyte
neuMono: Neutrophil-like monocyte
NK: Natural killer
PBMC: Peripheral blood mononuclear cell
PCA: Principal component analysis
PECAM1: Platelet/endothelial cell adhesion molecule 1
ROS: Reactive oxygen species
scRNA-seq: Single-cell RNA sequencing
scTCR-seq: Single-cell TCR sequencing
SEMA4: Semaphorin 4
SIRP: Signal regulatory protein
T2DM: Type 2 diabetes mellitus
Tcm: Central memory T cell
TCR: T-cell receptor
TdT: Terminal deoxynucleotidyl transferase
Tem: Effector memory T cell
Tex: Exhausted T cell
TGF-β: Transforming growth factor beta
Tdiff: Differentiated T cell
Tn: Naïve T cell
TNF: Tumor necrosis factor
Tpex: Precursor exhausted T cell
Tpre: Precursor T cell
Treg: Regulatory T cell
transB: Transitional B cell
TRA: T-cell receptor alpha chain
TRB: T-cell receptor beta chain
UMAP: Uniform Manifold Approximation and Projection
UMI: Unique molecular identifier
