# Supplementary material for: Integrated single-cell multi-omics analysis delineates neonatal immune dysregulation associated with gestational diabetes mellitus
Source: Front Immunol. 2026 Sep 30;17:1924940. doi: 10.3389/fimmu.2026.1924940 (PMC13661941; doi:10.3389/fimmu.2026.1924940)
Supplement: Supplementary file 1 [file DataSheet1.docx]

**Supplementary Material**

*Supplementary material is not typeset. Please ensure all information is clearly presented without tracked changes or line numbers, and that appropriate captions are included.*

# Supplementary Figures

**Supplementary Figure 1.** Quality control workflow and evaluation for the integrated single-cell transcriptomic dataset. **(a)** Violin plots displaying the distribution of quality control metrics for each sample: 500 < nFeature_RNA < 4,000; nCount_RNA < 15,000; MT% < 10%. **(b)** Scatter plots showing the correlations between nCount_RNA and MT% (left) and between nCount_RNA and nFeature_RNA (right) after quality control. **(c)** UMAP visualization of cell-type distribution across the in-house dataset and the external public dataset (GSE157007), used to assess the effect of batch correction. **(d)** Dot plot showing canonical marker gene expression for cell-type annotation. **(e) S**tacked bar plot showing the relative abundance of immune cell subsets across sample groups after excluding neutrophil populations.

**Supplementary Figure 2.** V-J gene combination distributions across individual samples and clonotype sharing between paired mother-neonate samples. **(a)** Stacked bar plot showing the proportional representation of the top 8 TRBV-TRBJ gene combinations in each individual sample. **(b)** Venn diagrams showing the overlap of the top 20 TCR VJ gene combinations among sample groups within each GDM mother-neonate pair.

**Supplementary Figure 3.** Tpex-like cell-specific transcriptional regulatory network. **(a)** Venn diagram showing the source intersection of regulons characteristic of the Tpex-like phenotype**. (b)** Network plot showing the transcriptional regulatory relationships of the regulons identified from the three-way intersection. **(c)** Distribution of Tpex associated gene set signature scores across pseudotime. Four panels are presented: top left, Tdiff cells; top right, Tpre cells; bottom left, Tpex like cells; bottom right, all T cells included in pseudotime reconstruction. Black filled circles labelled “1” denote trajectory branch points, and black dashed arrows indicate the inferred pseudotime progression direction. **(d)** Gene expression dynamics along pseudotime for selected stemness and exhaustion associated genes. Each dot represents an individual cell colour coded by its corresponding phenotype. The black solid line denotes the LOESS smoothed global trend of gene expression across all cells along the pseudotime axis

**Supplementary Figure 4.** Single-cell transcriptomic atlas of monocytes, NK cells, and B cells in the integrated dataset. **(a)** UMAP visualization of monocyte, NK cell, and B cell subsets. **(b)** Dot plot showing canonical marker gene expression. **(c)** Dot plot showing the top 3 enriched pathways for each subset. **(d)** Stacked bar plot showing the relative abundance of subsets across sample groups.
